# Postoperative neck tilt in Lenke 1 and 2 AIS patients after correction surgery: a novel predictive index

**DOI:** 10.1186/s12891-019-2792-9

**Published:** 2019-09-04

**Authors:** Yilin Yang, Mingyuan Yang, Zongde Yang, Kai Chen, Jinyi Bai, Jian Zhao, Haijian Ni, Changwei Yang, Ming Li

**Affiliations:** 1 Department of Orthopedics, Changhai Hospital, Second Military Medical University, Shanghai, People’s Republic of China; 2 Department of Orthopedics, Shanghai 10th people’s hospital, Tongji University, Shanghai, People’s Republic of China

**Keywords:** Postoperative neck tilt, Risk factors, Predictive index

## Abstract

**Background:**

Postoperative neck tilt (PNT) is a phenomenon in adolescent idiopathic scoliosis (AIS) patients which is distinct form shoulder imbalance. There were scarce studies performed to explore the risk factors for PNT in Lenke 1 and 2 AIS patients, and whether it can be predicted after surgery remains unknown. The objective of this study is to explore the prevalence and risk factors for PNT, and introduce an index for prediction of PNT in Lenke 1 and 2 AIS patients after correction surgery.

**Methods:**

Medical records of Lenke 1 and 2 AIS patients who received correction surgery were reviewed from February 2013 to February 2015. Posteroanterior films were evaluated before surgery and at 2 years’ follow-up. Patients were divided into two groups according to whether PNT occurred at the 2 years’ follow-up. Risk factors of PNT were analyzed, and PNT Index was proposed and verified.

**Results:**

One hundred two Lenke 1 and 2 AIS patients were recruited in this study. The prevalence of PNT after correction was 40.2%. According to the postoperative CAT (Cervical Axis Tilt), patients were divided into two group: PNT group (CAT≧5°, *n* = 41) and non-PNT group (CAT< 5°, *n* = 61). Postoperative T1 tilt, preoperative proximal thoracic curve (PTC), postoperative PTC and postoperative coronal balance (CB) were significantly different between two groups. Logistic regression showed that postoperative PTC and postoperative CB were the primary risk factors for PNT, which could be predicted by the regression equation: PNT Index = 1.1 x postoperative PTC (degrees) - 0.9 x postoperative CB (millimeters). On the basis of ROC curve, if PNT Index was more than 10, the occurrence rate of PNT was 86%. On the contrary, the rate of no PNT phenomenon was 80%.

**Conclusion:**

Postoperative PTC and postoperative CB were the important factors for PNT in Lenke 1 and 2 AIS patients. Sufficient correction of PTC and moderate correction of CB should be recommended when operating on Lenke1 and 2 AIS patients.

## Background

Adolescent idiopathic scoliosis (AIS) is a three-dimensional deformity of the spine that is characterized by axial rotation, sagittal plane deformity with thoracic hypokyphosis and translation of the spine in the coronal plane [1]. The overall prevalence of AIS is reported to be 0.47 to 5.2% in the current literature, which leads to great burdens to both families and society [2].

Up to date, correction surgery is still considered as an effective method to treat AIS, which aims to achieve correction of deformity, restore coronal and sagittal alignment, prevent further progression, and improve patients’ HRQOL (health-related quality of life) [2]. With constant development in newer techniques and instrumentation, great improvements have been achieved in correction rate of AIS, especially using pedicle screws that enable shorter fusion with more correction force [3, 4]. Recently, shoulder imbalance is getting increasing recognition of importance as this disastrous complication is observed commonly in AIS patients after correction surgery, which has great influence on patients’ appearance and HRQOL. On the one hand, the strength of pedicle screws instrumentation can correct the curve in three-dimension plane. On the other hand, this kind of internal fixation can frequently result in overcorrection, especially for the main thoracic curve (MTC), which can result in shoulder imbalance because the proximal thoracic curve (PTC) fails to compensate when fusing to upper thoracic segment [5].

Recently, according to the comparison of the characteristics between shoulder asymmetry (clavicle tilt and trapezial prominence) and radiographical parameters, Ono et al. [6] introduced the concept of ‘medial’ shoulder imbalance, which was confirmed to be a totally different entity from the conventional concept of ‘lateral’ shoulder imbalance [6]. Kwan et al. [7] reported that neck tilt with ‘medial’ shoulder imbalance was distinct from shoulder imbalance, and clinical neck tilt had been demonstrated poorly correlated with clinical shoulder imbalance.

In our clinical performance, postoperative neck tilt (PNT) is a common complication occurred in AIS patients after correction surgery. Like shoulder imbalance, PNT also has great influence on patients’ appearance and clinical outcomes. To avoid this disastrous complication and improve patients’ clinical outcomes and satisfaction, it is necessary to explore risk factors associated with PNT. However, there were scarce available literatures exploring the risk factors for PNT. Therefore, the aim of this study is to explore the risk factors for PNT and introduce an index for prediction of PNT in Lenke 1 and 2 AIS patients after correction surgery.

## Methods

### Patient population

Lenke1 and 2 AIS patients with posterior all-pedicle screw instrumentation in our center from February 2013 to February 2015, were retrospectively analyzed. The inclusion criteria were as follows: (a) Lenke 1 or 2 AIS patients with ages of 11 to 19 years; (b) MTC Cobb>40^o^; (c) one-stage posterior pedicle screw instrumentation was performed by the same treatment group; (d) sufficient full spine X-ray films before and after surgery. All patients included were followed up for at least 2 years. Other scoliosis, such as neuromuscular scoliosis and degenerative scoliosis, and patients without sufficient radiological parameters were excluded. This study was approved by the Institutional Review Board of our university, and all patients in our study provided written informed consent for the study.

### Data collection

Demographic data, including age, Risser sign, gender and Lenke types were recorded. The standing posteroanterior x-ray films were carried out while maintaining the neck and head in neutral relaxed position, and recorded preoperatively, postoperatively and at last follow-up, respectively. Coronal parameters, including CAT (cervical axis tilt), T1 tilt, PTC, MTC, TL/LC (thoracolumbar/lumbar curve), AVT (translation of apex vertebrae) of curves and CB (coronal balance) were measured and analyzed. PTC, MTC and TL/LC were measured using Cobb method. CAT was used to quantitatively evaluate the PNT, which is defined as the angle between the line drawn from the center of C7 to the center of C2 and plumb line [7]. T1 tilt represented an angle between the horizontal line and the line through the upper endplate of T1 [7]. AVT was used to measure the distance between apex vertebra of curve and CSVL (center sacral vertical line).CA (Clavicle angle: angle between the line connecting the lateral end of both clavicle with the horizontal plane) and RSH (Radiographic shoulder height: difference between the horizontal lines of the level of soft tissue shadow above the acromioclavicular joint) were measured to evaluate the shoulder balance. UIV-T1 was defined as the vertebra segments between UIV (Upper Instrumented Vertebrae) and T1. SRS-22 scores (Scoliosis Research Society questionnaire) were also evaluated to pay attention to the patient-centered outcome, including pain, appearance, activity, mental health, and satisfaction. All the parameters were obtained by two independent surgeons.

Patients were divided into two groups: PNT group (CAT≧5^o^) and non-PNT group (CAT<5^o^), according to the CAT 2-years postoperatively. 5^o^ was used as the threshold value because traditionally, a value of 5° has been accepted as measurement variation between measurements [8]. All the demographic, coronal and shoulder balance parameters were compared between these two groups (univariate analysis). Binary logistic regression analysis was also performed to detect the risk factors for occurrence of PNT phenomenon using the variables that were found significant in univariate analysis. Furthermore, PNT Index was set according to the results of logistic regression, as a novel predictor for PNT. The sensitivity and specificity of the predictive power of the occurrence of PNT phenomenon using PNT Index were calculated and the receiver operating characteristic (ROC) curve was drawn.

### Statistical analysis

The software package SPSS 22.0 was used for all statistical analysis. We used the methodology previously described by our former study [9]. Descriptive statistics were listed in the form of mean and standard deviation (SD), and categorical data were listed in numbers. Paired sample t test was used to compare the preoperative coronal and shoulder balance parameters and those at final follow-up. Independent two-sample t test was used to compare the differences of variables between two groups. X^2^ test was used to compare the differences of count data. Binary logistic regression models, with forward elimination (Conditional), were constructed using variables that were found significant in a comparison study in order to find independent risk factors associated with PNT. ROC curves were constructed to detect the optimal cut-off value of PNT Index as indicators for occurrence of PNT phenomenon. *P* < 0.05 was considered with significant difference.

## Results

### General information

A total of 102 Lenke 1 and 2 AIS patients (male/female: 35/67) were recruited in our study, with the mean age of 14.76 years (10–19 years). There were 72 Lenke 1 patients and 30 Lenke 2 patients. The number of segments between UIV and T1 as 0, 1, 2, 3, 4 and 5 was 7, 10, 36, 36, 11 and 2, respectively. All the demographics data and radiological parameters were shown in Table 1.

**Table 1:** The general characteristics of included patients

Variables	Minimum	Maximum	Mean	SD
Demographic parameters				
Age (years)	10.00	19.00	14.76	2.04
Risser sign (^o^)	0.00	5.00	3.30	1.46
Gender (male/female)	35/67			
Lenke types (n)				
1A−/1AN/1A+/1B−/1BN/1CN	8/28/7/6/11/12			
2A−/ 2AN/2BN/2C−/2CN	6/10/8/3/3			
UIV-T1 (segment) (0/1/2/3/4/5) (n)	7/10/36/36/11/2			
Coronal parameters				
Preoperative CAT (^o^)	−8.00	20.00	3.28	3.57
Postoperative CAT (^o^)	−6.00	9.00	3.36	2.99
Preoperative T1 tilt (^o^)	−18.40	26.00	4.85	5.49
Postoperative T1 tilt (^o^)	−6.00	14.00	4.71	3.69
Preoperative PTC (^o^)	3.00	63.00	27.31	10.23
Postoperative PTC (^o^)	2.00	38.00	18.38	8.55
Preoperative AVT of PTC (mm)	1.00	46.00	21.07	15.18
Postoperative AVT of PTC (mm)	2.00	15.00	7.60	4.27
Preoperative MTC (^o^)	18.00	104.00	48.15	11.87
Postoperative MTC (^o^)	3.00	57.00	19.73	9.71
Preoperative AVT of MTC (mm)	36.00	74.00	51.24	11.84
Postoperative AVT of MTC (mm)	5.00	350	15.02	8.41
Preoperative TL/LC (^o^)	5.00	58.00	28.80	11.45
Postoperative TL/LC (^o^)	1.00	27.00	12.08	6.72
Preoperative AVT of TL/LC (mm)	0.00	23.00	7.15	5.13
Postoperative AVT of TL/LC (mm)	0.00	19.00	7.79	4.93
Preoperative CB (mm)	−16.35	40.00	11.94	9.70
Postoperative CB (mm)	−14.00	25.00	7.48	6.90
Shoulder balance				
Preoperative CA (^o^)	−10.00	11.00	1.89	2.69
Postoperative CA (^o^)	−2.00	8.00	1.93	1.55
Preoperative RSH (mm)	−24.00	40.00	8.18	10.96
Postoperative RSH (mm)	−5.00	24.00	7.49	5.73
HRQOL				
Preoperative SRS-22 total score	3.20	4.40	3.92	0.25
Postoperative SRS-22 total score	3.40	4.40	4.08	0.22

As shown in Table 2, PTC, MTC, TL/LC, AVT, CB and SRS-22 changed significantly after correction surgery (all *P* < 0.001); while no significant difference of CAT, T1 tile, CA and RSH was observed between pre-operation and immediate post-operation (all *P* > 0.05). Moreover, postoperative CAT (6.55 ± 1.19°vs 5.33 ± 1.41°, *P* = 0.001), postoperative T1 tilt (6.02 ± 3.46°vs4.34 ± 4.02°, *P* < 0.001), postoperative AVT of PTC (10.29 ± 3.14 mm vs 9.13 ± 2.86 mm, *P* = 0.038), postoperative TL/LC (10.63 ± 6.18°vs14.90 ± 8.17°, *P* = 0.002) patients in postoperative neck tilt group changed significantly during at least two-year follow-up, while no significant difference of the other coronal and shoulders balance parameters in postoperative neck tilt group and all measured radiological parameters in postoperative non-neck tilt group was observed between post-operation and last follow-up (all *P* > 0.05).

**Table 2:** Comparisons of coronal and shoulder balance parameters between pre-operation and post-operation in AIS

Variables	Pre-operation	Post-operation	*P* value
Coronal parameters			
CAT (^o^)	3.28 ± 3.57	3.36 ± 2.99	0.839
T1-tilt (^o^)	4.85 ± 5.49	4.71 ± 3.69	0.774
PTC (^o^)	27.31 ± 10.23	18.38 ± 8.55	< 0.001
AVT of PTC (mm)	21.07 ± 15.18	7.60 ± 4.27	< 0.001
MTC (^o^)	48.15 ± 11.87	19.73 ± 9.71	< 0.001
AVT of MTC (mm)	51.24 ± 11.84	15.02 ± 8.41	< 0.001
TL/LC (^o^)	28.80 ± 11.45	12.08 ± 6.72	< 0.001
AVT of TL/LC (mm)	7.15 ± 5.13	4.79 ± 4.93	< 0.001
CB (mm)	11.94 ± 9.70	7.48 ± 6.90	< 0.001
Shoulder balance			
CA (^o^)	1.89 ± 2.69	1.93 ± 1.55	0.887
RSH (mm)	8.18 ± 10.96	7.49 ± 5.73	0.529
HRQOL			
SRS-22	3.92 ± 0.25	4.08 ± .022	< 0.001

### Univariate analysis

Forty-one patients suffered from PNT (CAT≧5^o^) 2 years after correction surgery, and were recruited in PNT group. No significant PNT was observed in 61 AIS patients, and were recruited in non-PNT group. The prevalence of PNT in AIS patients at 2-year follow-up was 40.2%. Significant differences of parameters between these two groups were found as follows: postoperative T1 tilt (6.02 ± 3.46°vs 3.82 ± 3.60°, *P* = 0.003), preoperative PTC (31.56 ± 8.06°vs 24.46 ± 10.59°, *P* < 0.001), postoperative PTC (22.76 ± 7.87°vs 15.44 ± 7.73°, *P* < 0.001), postoperative AVT of PTC (10.29 ± 3.14 mm vs 5.71 ± 3.95, *P* < 0.001) and immediate postoperative CB (4.32 ± 5.86 mm vs 9.61 ± 6.76 mm, *P* < 0.001). The data are summarized in Table 3. However, no significant difference of age, Risser sign, gender, UIV-T1, preoperative CAT, preoperative T1 tilt, preoperative AVT of PTC, preoperative MTC, postoperative MTC, preoperative AVT of MTC, postoperative AVT of MTC, preoperative TL/LC, postoperative TL/LC, preoperative AVT of TL/LC, postoperative AVT of TL/LC, preoperative CB, preoperative CA, postoperative CA, preoperative RSH, and preoperative RSH (all *P* > 0.05). The data are summarized in Table 3.

**Table 3:** Comparisons of demographic, coronal and shoulder balance parameters between PNT group (CAT≧5^o^) and non-PNT group (CAT<5^o^)

Variables	Postoperative neck tilt group CAT≧5^o^ (*n* = 41)	Postoperative non-neck tilt group CAT<5^o^ (*n* = 61)	*P* value
Demographic parameters			
Age (years)	14.81 ± 2.44	14.74 ± 1.78	0.897
Risser sign (^o^)	3.12 ± 1.66	3.43 ± 1.31	0.305
Gender (male/female)	15/26	20/41	0.692
UIV-T1 (segment) (0/1/2/3/4/5) (n)	3/4/18/9/7/0	4/6/18/27/4/2	0.118
Coronal parameters			
Preoperative CAT (^o^)	3.78 ± 3.21	2.95 ± 3.78	0.252
Preoperative T1 tilt (^o^)	5.16 ± 6.31	4.64 ± 4.90	0.640
Postoperative T1 tilt (^o^)	6.02 ± 3.46	3.82 ± 3.60	0.003
Preoperative PTC (^o^)	31.56 ± 8.06	24.46 ± 10.59	< 0.001
Postoperative PTC (^o^)	22.76 ± 7.87	15.44 ± 7.73	< 0.001
Preoperative AVT of PTC (mm)	24.28 ± 14.07	18.82 ± 15.63	0.073
Postoperative AVT of PTC (mm)	10.29 ± 3.14	5.71 ± 3.95	< 0.001
Preoperative MTC (^o^)	48.34 ± 9.39	48.02 ± 13.36	0.893
Postoperative MTC (^o^)	17.85 ± 8.07	20.98 ± 10.55	0.111
Preoperative AVT of MTC (mm)	53.81 ± 9.85	49.43 ± 12.83	0.066
Postoperative AVT of MTC (mm)	15.52 ± 7.94	14.67 ± 8.78	0.615
Preoperative TL/LC (^o^)	28.19 ± 12.93	29.21 ± 10.43	0.662
Postoperative TL/LC (^o^)	10.63 ± 6.18	13.05 ± 6.94	0.075
Preoperative AVT of TL/LC (mm)	7.21 ± 5.14	7.10 ± 6.22	0.819
Postoperative AVT of TL/LC (mm)	8.01 ± 5.67	7.66 ± 4.10	0.743
Preoperative coronal balance (mm)	12.63 ± 11.66	11.48 ± 8.20	0.560
Postoperative coronal balance (mm)	4.32 ± 5.86	9.61 ± 6.76	< 0.001
Shoulder balance			
Preoperative CA (^o^)	1.29 ± 2.26	2.30 ± 2.90	0.062
Postoperative CA (^o^)	1.76 ± 1.51	2.05 ± 1.58	0.352
Preoperative RSH (mm)	8.37 ± 8.35	8.05 ± 12.47	0.887
Postoperative RSH (mm)	6.80 ± 5.61	7.95 ± 5.81	0.325

### Multivariate analysis

Results of binary logistic regression showed that the postoperative PTC and postoperative CB were the primary factors included in the equation [Odds Ratio (OR) = 1.1, and 0.894, respectively], while other variables were not included in the regression equation (preoperative PTC: *P* = 0.883 and postoperative T1 tilt: *P* = 0.159), as indicated in Table 4.

**Table 4:** Binary logistic regression analysis for risk factors of PNT

Variables	B	S.E.	Wald	Df	*P* value	Exp (B)	95% CI	
							Lower	Upper
Postoperative PTC	0.28	0.041	5.486	1	0.019	1.100	1.03	1.41
Postoperative CB	0.112	0.041	7.427	1	0.006	0.894	0.69	0.93
Constant	1.806	0.867	4.336	1	0.037	0.164		

**Fig. 1:**
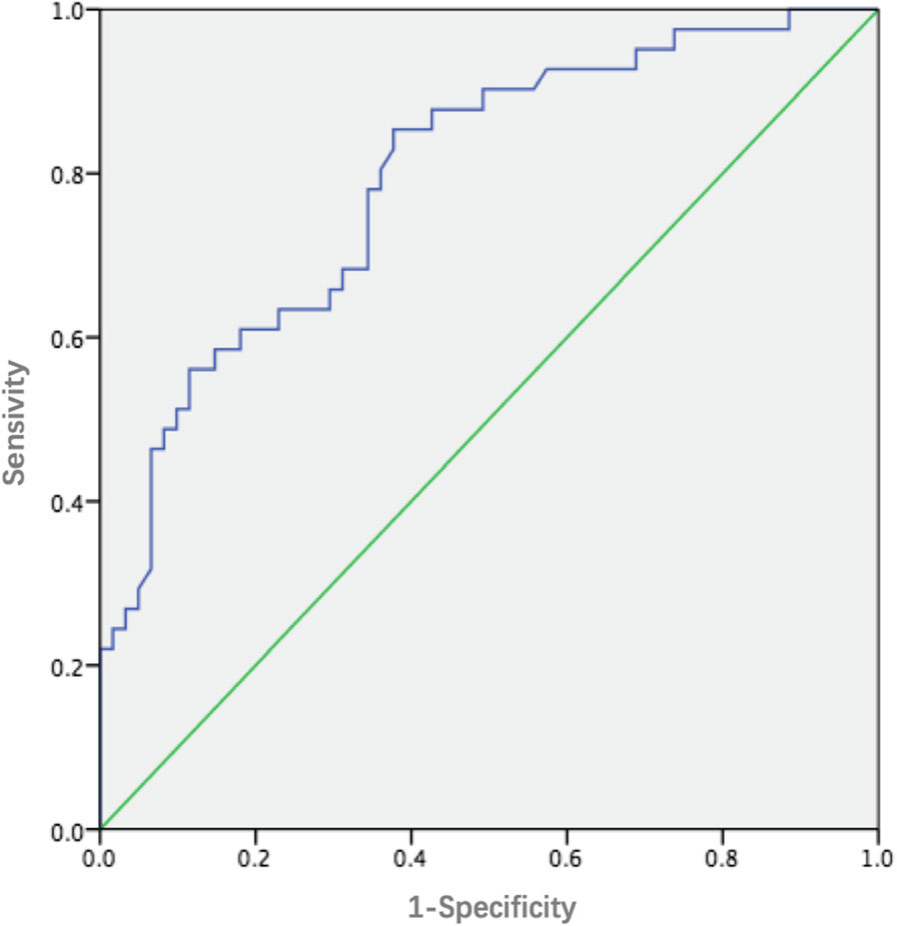
ROC curve of predictive power of the occurrence of PNT using PNT Index

According to the results of regression equation, we defined the PNT Index as 1.1 x postoperative PTC (degrees) - 0.9 x postoperative CB (millimeters). On the basis of the ROC curve, the optimal cut-off values of PNT Index as indicators for occurrence of PNT were projected to be 10 (Fig. 1). If this index was beyond 10, the occurrence rate of PNT was 86%. On the contrary, the rate of no PNT was 80%.

**Fig. 2:**
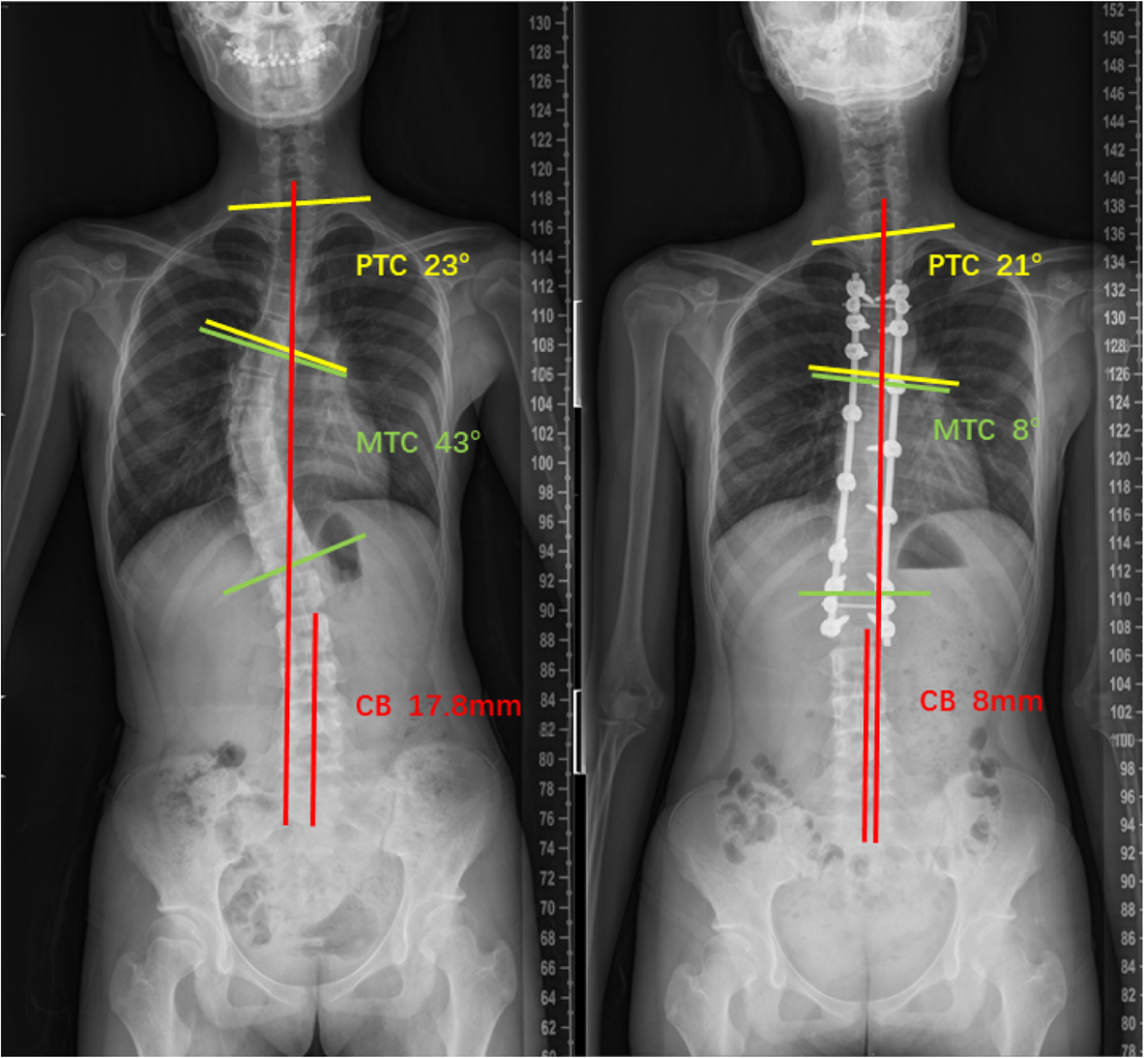
A 13-years old AIS patient received correction surgery in August 2013, and she suffered from PNT at 2 years follow-up. The postoperative PTC and CB was 25^o^ and 10 mm, respectively. The PNT Index was 18.5 according to the regression equation

A typical case was represented in Fig. 2. A 16-years old Lenke 1 AIS patient received correction surgery in July 2011, and she suffered from PNT at 2 years follow-up. The postoperative PTC and CB was 21^o^ and 8 mm, respectively. The PNT Index was 15.9 according to the regression equation.

In the at least 2-year follow-up period, 41 Lenke 1 and 2 AIS patients in postoperative neck tilt group exhibited significant decrease in postoperative CAT (6.55 ± 1.19°vs 5.33 ± 1.41°, *P* = 0.001), postoperative T1 tilt (6.02 ± 3.46°vs 4.34 ± 4.02°, *P* < 0.001), Postoperative AVT of PTC (10.29 ± 3.14 mm vs 9.13 ± 2.86 mm, *P* = 0.038), and significant increase in postoperative TL/LC (10.63 ± 6.18°vs14.90 ± 8.17°, *P* = 0.002), which indicated that Lenke 1 and 2 AIS patients with PNT showed spontaneous correction during follow-up. For patients in postoperative non-neck tilt group, coronal and shoulder radiographic parameters remained balanced during follow-up period without significant change (all *P* > 0.05), and all the data were shown in Table 5.

**Table 5:** Comparisons of radiological parameters and SRS-22 scores between immediate post-operation and last follow-up in Lenke 1 and 2 AIS patients with and without PNT

Variables	Immediate post-operation	Last follow-up	*P* value
Postoperative neck tilt group (*n* = 41)			
Coronal Parameters			
Postoperative CAT (°)	6.55 ± 1.19	5.33 ± 1.41	0.001
Postoperative T1 tilt (°)	6.02 ± 3.46	4.34 ± 4.02	< 0.001
Postoperative PTC (°)	22.76 ± 7.87	24.22 ± 8.11	0.307
Postoperative AVT of PTC (mm)	10.29 ± 3.14	9.13 ± 2.86	0.038
Postoperative MTC (°)	17.85 ± 8.07	19.01 ± 8.52	0.123
Postoperative AVT of MTC (mm)	15.52 ± 7.94	15.23 ± 8.89	0.616
Postoperative TL/LC (°)	10.63 ± 6.18	14.90 ± 8.17	0.002
Postoperative AVT of TL/LC (mm)	8.01 ± 5.67	7.75 ± 6.02	0.441
Postoperative coronal balance (mm)	4.32 ± 5.86	3.62 ± 4.04	0.146
Shoulder balance			
Postoperative CA (^o^)	1.76 ± 1.51	1.45 ± 1.18	0.251
Postoperative RSH (mm)	6.80 ± 5.61	6.45 ± 4.87	0.222
Postoperative non-neck tilt group (*n* = 61)			
Coronal Parameters			
Postoperative CAT (°)	2.74 ± 1.56	3.01 ± 1.52	0.388
Postoperative t T1 tilt (°)	3.82 ± 2.60	4.25 ± 3.19	0.551
Postoperative PTC (°)	15.44 ± 7.73	17.21 ± 6.34	0.275
Postoperative AVT of PTC (mm)	5.71 ± 3.95	5.22 ± 3.72	0.761
Postoperative MTC (°)	20.98 ± 10.55	22.37 ± 8.67	0.430
Postoperative AVT of MTC (mm)	14.67 ± 8.78	12.41 ± 7.48	0.178
Postoperative TL/LC (°)	13.05 ± 6.94	15.18 ± 5.17	0.095
Postoperative AVT of TL/LC (mm)	7.66 ± 4.10	7.31 ± 5.89	0.796
Postoperative coronal balance (mm)	9.61 ± 6.76	7.83 ± 7.78	0.064
Shoulder balance			
Postoperative CA (°)	2.05 ± 1.58	1.12 ± 1.09	0.131
Postoperative RSH (mm)	7.95 ± 5.81	6.40 ± 3.19	0.072

## Discussion

Neck tilt, first reported by Kwan et al. [7], is a novel concept which is distinct from the conventional shoulder imbalance. In their study, Kwan et al. [7] investigated the differences between neck tilt and shoulder imbalance in AIS patients based on their clinical and radiological characteristics, and established a brand new clinical neck tilt grading system as blow: I). Grade 0: no neck tilt. II). Grade 1: correctable neck tilt with active neck muscle contraction, and equal trapezius muscle height. III). Grade 2: uncorrectable neck tilt, whereas trapezius muscle height difference less than 1 cm. IV). Grade 3: uncorrectable neck tilt, and trapezius muscle height difference more than 1 cm. This new neck tilt grading has been demonstrated with strong interobserver and intraobserver reliability. Furthermore, they found that CHD (coracoid height difference), CRID (clavicle\rib intersection distance), CA and RSH were strongly correlated with clinical shoulder height, while CAT and T1 tilt had a good correlation with clinical neck tilt grading. However, there was poor correlation between clinical neck tilt grading and clinical shoulder height. Bago’s [10] and Akel’s study [11] also showed that T1 tilt had the poorest correlation with clinical shoulder appearance when compared to the other shoulder radiological parameters such as CHD, CRID and CA. All these findings indicated that ‘neck tilt’ was a distinct concept compared with ‘shoulder imbalance’.

Our univariate analysis showed that there was significant difference of postoperative T1 tilt between patients with and without PNT. However, we did not observe any significant difference of CA and RSH between these two groups. Our findings further verified Kwan’s [7], Akell’s [11] and Bago’s results [10] that neck tilt is distinct from shoulder imbalance. Postoperative T1 tilt in PNT group was significantly larger than that in non-PNT group (6.02 ± 3.46 vs. 3.82 ± 3.60, *P* = 0.003). T1 vertebrae constructs the base of the neck and neck tilt will result in tilting of the T1 vertebrae [7]; therefore, it is easily understood why larger postoperative T1 tilt was observed in AIS patients with PNT. PNT occurs more frequently in AIS patients with rigid PTC, which is supported by our findings that larger preoperative, postoperative PTC and postoperative AVT of PTC were observed in PNT group than those in non-PNT group. On the other hand, improper treatment of PTC may also be important contributor to neck tilt in AIS patients after correction surgery. Furthermore, Kwan’s thought that overcorrection of the MTC would result in an uncompensated PTC, which would lead to neck tilt as well. However, no significant difference of preoperative and postoperative MTC was observed between PNT group and non-PNT group in our study, suggesting that correction of MTC might not play an important role in the occurrence of PNT. Therefore, how to correct the PTC and MTC of AIS, especially in AIS patients with rigid curves during the surgical procedures should be further investigated.

Our study also indicated that postoperative CB was also significant different between PNT group and non-PNT group. Compared with patients in no-PNT group, AIS patients with PNT tended to have smaller postoperative CB. In our opinion, neck tilt may be a compensatory mechanism for coronal balance. Overcorrection of coronal alignment may lead to the tendency of coronal imbalance. The lumbar curves possess limited ability of compensation for coronal imbalance because of the long fusion segments with few reserve motion segments. Consequently, neck tilt occurs to compensate the whole coronal alignment, especially in patients with rigid PTC. Therefore, to decrease the incidence of PNT, CB should be moderately corrected and the remaining MTC and TL/LC gives rise to the compensation of reserve motion to avoid the occurrence of this complication. It is important to take into consideration the correction rate of coronal alignment during the surgery. Thus, we recommend moderate correction of coronal alignment, aiming to restore the whole coronal balance and avoid the occurrence of neck tilt after correction surgery.

The multivariate analysis in this study indicated that the main factors influencing the occurrence of PNT were postoperative PTC and postoperative CB, while other variables were not primary contributors (all *P* > 0.05). It indicated that if the remaining PTC after correction surgery were too large, while the postoperative CB was relatively small (overcorrection of coronal alignment), the possibility of the occurrence of PNT would be increased.

For Lenke 1 and 2 AIS patients, how to choose the upper fusion level and correct the PTC remains controversial [12–14]. The aims of PTC correction in AIS patients are to restore the coronal alignment and avoid the shoulder imbalance as well. Based on our study, avoidance of PNT should also be added into the aims of PTC correction, and taken into consideration during the decision-making and surgery. The logistic regression showed that postoperative PTC and postoperative CB were the primary risk factors for PNT, and development the regression equation: PNT Index = 1.1 x postoperative PTC (degrees) - 0.9 x postoperative CB (millimeters). Based on our findings, it can be noticed that the reason of PNT in Lenke 1 and 2 AIS patients is due to the too much correction of CB and inadequate correction of PTC. Thus, moderate correction of PTC and CB should be taken into consideration during the surgery. ROC curve showed that the threshold value of PNT was 10. On the basis of ROC curve, if PNT Index was more than 10, the occurrence rate of PNT was 86%. On the contrary, the rate of no PNT phenomenon was 80%. In practice, for AIS patients with rigid PTC, PTC may not change significantly compared with preoperative PTC, even if pedicle screws are used. In order to prevent PNT in these patients, the minimum postoperative CB could be calculated according to preoperative PTC [(1.1xpreoperative/postoperative PTC-10)/0.9]. For AIS with flexible PTC, sufficient correction of PTC and moderate correction of CB should be considered. The calculated PNT Index should be less than 10 according to the regression equation using postoperative PTC and CB, then the incidence of PNT could be effectively decreased.

In the at least two-year follow-up, although Lenke 1 and 2 AIS patients with PNT showed spontaneous improvement during follow-up, there was still 40.2% patients exhibited PNT whose CAT could not be restored at final follow-up, which might influence patients’ appearance and HRQOL. Therefore, the explorations of risk factors for postoperative neck tilt in Lenke 1 and 2 AIS patients still needs investigations for the following reasons: 1). Patients with PNT had worse cosmetic appearance, which lead to worse satisfaction; 2). Not all the Lenke 1 and 2 patients with PNT could be spontaneous corrected during follow-up; therefore, spinal surgeons should try their best to avoid postoperative neck tilt when treating Lenke 1 and 2 AIS patients to prevent the occurrence of neck tilt at final follow-up and to improve patients’ HRQOL as well. 3). Our study also suggested that patients with acceptable CAT who did not present neck tilt at final follow-up, also laying the importance of restoration of neck alignment restoration at immediate post-operation.

Although we found a novel predictor for postoperative neck tilt phenomenon and evaluate its effectiveness, there are several limitations should be addressed. First, the present study was based on a single spine surgery center and the sample size was relatively limited. Second, studies with further long follow-up should be performed to detect the effectiveness of PNT Index. Third, Kwan’s study [15] also found that UIV tilt angle might contribute to neck tilt with ‘medial’ shoulder imbalance, which was not studied in our study. Therefore, large-scale and multicenter studies should be performed to make a more comprehensive research into the risk factors for PNT as well as the effectiveness of PNT Index in predicting the neck tilt phenomenon in AIS patients after correction surgery.

## Conclusion

The prevalence of PNT in AIS patients after correction was 40.2%. PNT group and non-PNT group showed significant differences in postoperative T1 tilt, preoperative PTC, postoperative PTC, postoperative AVT of PTC and postoperative CB. Whereas postoperative PTC and postoperative CB were the primary factors for PNT. Therefore, we recommended sufficient correction of PTC and moderate correction of CB when operating on Lenke1 and 2 AIS patients.

## Data Availability

The datasets generated and/or analyzed during the current study are not publicly available as they will be studied for further research, but are available from the corresponding author on reasonable request.

## Abbreviations

AIS: Adolescent idiopathic scoliosis
AVT: Translation of apex vertebrae
CA: Clavicle angle
CAT: Cervical axis tilt
CB: Coronal balance
CHD: Coracoid height difference
CRID: Clavicle\rib intersection distance
MTC: Main thoracic curve
OR: Odds ratio
PNT: Postoperative neck tilt
PTC: Proximal thoracic curve
ROC: Receiver operating characteristic
RSH: Radiographic shoulder height
SD: Standard deviation
SRS: Scoliosis research society questionnaire
TLC/LC: Thoracolumbar/lumbar curve
UIV: Upper Instrumented vertebrae
